# Shield as Signal: Lipopolysaccharides and the Evolution of Immunity to Gram-Negative Bacteria

**DOI:** 10.1371/journal.ppat.0020067

**Published:** 2006-06-30

**Authors:** Robert S Munford, Alan W Varley

**Affiliations:** Scripps Research Institute, United States of America

According to the innate immunity concept [1], animals defend themselves from microbes by recognizing pathogen-associated molecular patterns. To detect many Gram-negative bacteria, animals use the CD14–MD-2–TLR4 receptor mechanism to recognize the lipid A moiety of the cell wall lipopolysaccharide (LPS). Lipid A is a glucosamine disaccharide that carries phosphates at positions 1 and 4′ and usually has four primary (glucosamine-linked) hydroxyacyl chains and one or more secondary acyl chains. Gram-negative bacteria produce numerous variations on this basic structure, yet sensitive LPS recognition and pro-inflammatory signaling by human TLR4 occur only when lipid A has both phosphates and is hexaacyl, with two secondary acyl chains.

What might bacteria derive from producing this type of lipid A, and what do animals gain from recognizing it? A survey of diverse lipid A structures found that the best-recognized configuration is produced by most of the aerobic or facultatively anaerobic Gram-negative bacteria that can live in the gastrointestinal and upper respiratory tracts. We hypothesize that the CD14–MD-2–TLR4 mechanism evolved to recognize not just pathogens, but also many of the commensals (normal flora) and colonizers that can inhabit the body's most vulnerable surfaces. Producing this lipid A structure seems to favor bacterial persistence on host mucosae, whereas recognizing it allows the host to kill invading bacteria within subepithelial tissues and prevent dissemination. A conserved host lipase can then limit the inflammatory response by removing a key feature of the lipid A signal, the secondary acyl chains.

## Acylation of Lipid A: Strengthening the Shield?

Gram-negative bacteria that inhabit water, soil, plants, or insects display impressive diversity in their lipid A structures (see Table S1). Although the backbone is almost always a bisphosphorylated disaccharide that has three or more primary fatty acyl chains, the secondary acyl chains differ in their number, length, and degree of saturation. In contrast, the lipid A structures produced by most of the aerobic and facultatively anaerobic Gram-negative bacteria that live as human mucosal commensals, colonizers, or pathogens [2] are monotonously similar: they have two phosphates, four primary hydroxyacyl chains (3-hydroxymyrisate or 3-hydroxylaurate), and two saturated secondary acyl chains (laurate, myristate, or both); we shall refer to this composition as “mucosal” lipid A. Since these bacteria differ in many other ways, the fact that their lipid As are so similar suggests that this structure may confer some advantage.

**Figure 1:**
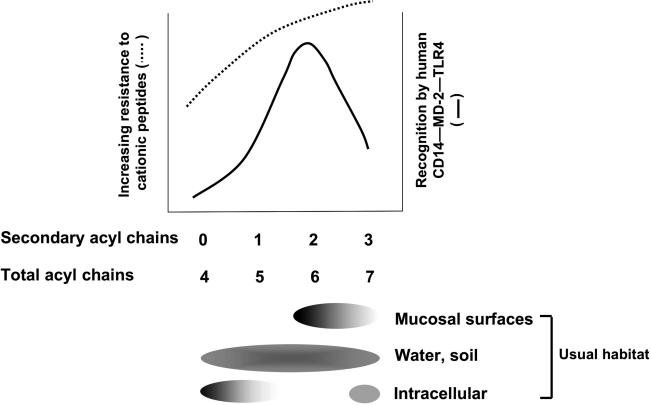
Lipid A Structure, Bacterial Habitat, and Host Recognition Approximate relationship between resistance to CAMPs, recognition by MD-2–TLR4, and lipid A acyl chain composition for Gram-negative aerobic or facultatively anaerobic bacteria living in different habitats. The number, length, and saturation of the acyl chains may all influence recognition by TLR4. In mucosal lipid A, the acyl chains are saturated and usually have 12 or 14 carbons.

Mucosal secretions contain numerous cationic antimicrobial peptides (CAMPs) [3]. As noted by Miller [4] and others, increased resistance to CAMPs and other host molecules may explain why Gram-negative bacteria that colonize mucosae usually make LPS with six or more acyl chains (Figure 1). Although we found no demonstration that this lipid A structure enables commensal bacteria to thrive on mucosal surfaces, the evidence that it does so for colonizers and pathogens is extensive. Having hexaacyl (rather than pentaacyl) lipid A enables Bordetella and Haemophilus species to persist in the respiratory tract [5–7] and Neisseria gonorrhoeae to survive within epithelial cells [8]. Pseudomonas aeruginosa lives in water, produces a predominantly pentaacylated LPS, and does not colonize the mucosae of normal humans. When P. aeruginosa colonize the airways of children with cystic fibrosis, however, the bacteria often adapt by producing hexaacylated (and even heptaacylated) lipid A [9]. Mucosal lipid A is also found in intestinal pathogens: Shigella and Salmonella, pathogenic Escherichia coli [10], Aeromonas species, Plesiomonas shigelloides, and Vibrio cholerae O1. In Salmonella and some others, a PhoP/PhoQ-regulated transcriptional program promotes lipid A palmitoylation (heptaacylation) along with other changes that increase resistance to CAMPs [4,11–13]. Other mucosal bacteria may also produce lipid A that is more hydrophobic than mucosal lipid A, with longer secondary chains (Campylobacter jejuni) or more of them: heptaacyl (Moraxella) or octaacyl (V. cholerae O139). Those that produce less hydrophobic lipid A seem to be special cases: the pentaacyl LPS of Chlamydia species is found in spore-like elementary bodies, and Helicobacter pylori, with tetraacyl LPS, is adapted to live in the stomach.

Many other disease-associated Gram-negative bacteria have nonmucosal habitats. Their lipid A moieties differ from the typical mucosal structure by having shorter or longer acyl chains, unsaturated acyl chains, only four or five chains, or only one phosphate (see Table S1): Legionella (water habitat, often in free-living amoebae), Burkholderia pseudomallei (soil and water), Yersinia pestis (small rodents, lice), Coxiella burnetti (intracellular, livestock), Leptospira (water, animal urine), and Francisella tularensis (ticks, rabbits, other small animals). These pathogens usually enter vertebrate tissues via insect bites or cuts, within inhaled droplets, or across the conjunctivae. Brucellae (livestock), which inhabit macrophages yet are typically acquired via ingestion, also produce a nonmucosal LPS [14].

## How Animals Sense Mucosal Gram-Negative Bacteria: Shield as Signal

Whereas bacterial peptide resistance and outer membrane impermeability seem to vary directly with the number of acyl chains, the inflammation-inducing CD14–MD-2–TLR4 sensory mechanism best recognizes lipid A that has the hexaacyl mucosal lipid A structure [15–20] (Figure 1). In support, Hajjar et al. [21] reported that a discrete extracellular region of human TLR4 enables recognition of hexaacyl, but not pentaacyl, P. aeruginosa LPS. Further discrimination is performed by MD-2 [22]. The same recognition pattern has been found for all mammals tested except rodents [23].

Evidence that lipid A structure influences the recognition of intact bacteria by host cells came from mutating enzymes that attach secondary acyl chains to the backbone. Somerville et al. [24] found an E. coli mutant that was unable to attach the secondary myristate at position 3′ and could not stimulate human endothelial cells. Having a hexaacyl LPS also enhances other responses to intact bacteria, including the induction of tumor necrosis factor by Salmonella in vivo [25], the initiation of intestine wall inflammation by S. flexneri [26], and the production of IL-8 by bladder epithelial cells infected with E. coli [27]. These and other studies convincingly showed that LPS is the major structure sensed by most host defense cells when they interact with bacteria that produce mucosal lipid A [28].

Although the TLR4-based mechanism for sensing LPS has been highly conserved [29], how it benefits the host is only partly understood. Gastrointestinal epithelial cells evidently do not express TLR4 on their lumenal surfaces under normal in vivo conditions, so it is unlikely that they sense LPS or Gram-negative bacteria in the fecal stream [30]. On the other hand, Rakoff-Nahoum et al. [31] recently found that subepithelial TLR4-dependent sensing protects damaged gut from injury by commensal bacteria. Perhaps the key function of this system is to sense bacteria as they enter submucosae, thus mobilizing defenses that confine bacterial invasion, and the inflammatory response to it, to the local site [32,33]. Shigella invasion through the colonic epithelium prompts intense local inflammation, for example; the fact that Shigella possess hexaacyl LPS may help the host confine infection to the intestine (bacteremia rarely occurs) [26,34]. TLR4-dependent responses to other enteric pathogens may also damage the intestinal wall, yet these bacteria also do not often spread to the bloodstream [35]. Similar local responses may help restrict disease caused by most Haemophilus and Bordetella species to the respiratory tract, most strains of N. gonorrhoeae to the urethral mucosa, and E. coli to the bladder [27]. In contrast, producing heptaacylated LPS may help Salmonellae [11] avoid recognition within the intestinal submucosa and grow in extraintestinal tissues.

Most mucosal Gram-negative bacteria that enter the bloodstream are rapidly killed. In many instances, LPS sensing is required for effective elimination [32,36]. Few Gram-negative bacteria grow to high density in the blood of immunocompetent humans [37]; of these, Y. pestis [38] and B. pseudomallei [39] produce lipid A structures that are poorly sensed by TLR4 [40–42]. As suggested by others [29,40,42], these bacteria may be effective human pathogens, at least in part, because the TLR4 mechanism does not recognize them. This notion may also apply to other bacteria that lack the mucosal lipid A structure and are weak TLR4 agonists: F. tularensis [43,44], L. pneumophila [45], C. burnetti [46], H. pylori [47], Brucellae [48,49], and Leptospira [50] species. According to this hypothesis, engineering these bacteria to produce mucosal lipid A should alter their ability to cause disease.

The most obvious exceptions are the pathogenic Neisseriae. Both N. meningitidis and N. gonorrhoeae produce a mucosal lipid A and colonize mucosal surfaces. How they invade the bloodstream is not known [51], but they usually seem to do so without triggering local inflammation. They illustrate the important point that the lipid A–TLR4 interaction is but one element of the confrontation between bacterial pathogen and animal host.

## Destroying the Signal: Acyloxyacyl Hydrolysis

**Figure 2:**
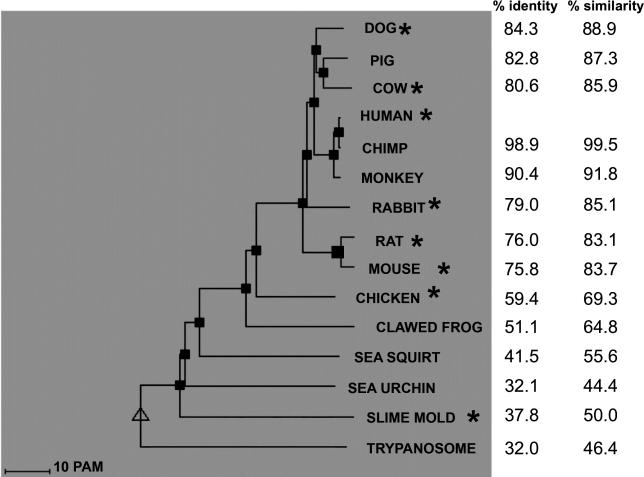
AOAH Phylogenetic Tree Topological algorithm derived using DisplayFam [69] analysis of available sequences. Amino acid similarity/identity to the full-length human sequence is shown (includes both subunits and the pro-peptide). AOAH-like sequences have not been found in fish or insects. The asterisk indicates that AOAH-like enzymatic activity has been demonstrated in one or more cell types. See Table S2 for accession numbers.

Whereas Dictyostelium discoideum produces several lipid A–deacylating enzymes, only one has been found in mammals. Acyloxyacyl hydrolase (AOAH) removes only the secondary chains from lipid A; it cleaves saturated, short secondary chains, as are found in the mucosal lipid A structure, more rapidly than it removes long unsaturated ones [52,53]. A phylogenetic analysis revealed high conservation for both the AOAH large subunit, which has the bacterial GDSL lipase motif [54], and the small subunit, a member of the saposin-like protein family [55] and the likely LPS recognition motif (Figure 2). Indeed, AOAH has evidently been more highly conserved than has TLR4 [29]

In vertebrates, AOAH is produced by neutrophils, dendritic cells, renal cortical epithelial cells, and monocyte-macrophages. AOAH treatment greatly reduces LPS sensing via TLR4 [52], and LPS may remain stimulatory for weeks in mice that cannot deacylate it [56]. AOAH thus can limit inflammatory responses to bacteria that produce mucosal lipid A. Deacylation occurs slowly, reaching completion after the early recognition phase of antibacterial innate immunity has occurred.

## Other Signals

The host response to LPS also has noninflammatory, immunostimulatory elements. Lipid A analogs that lack the optimal configuration for inducing inflammation may be excellent adjuvants, enhancing acquired immune responses in ways that mimic those induced by LPS itself [57,58]. The mucosal lipid A motif triggers inflammation (and toxicity), whereas adjuvanticity may also follow TLR4-based recognition of lipid A molecules that have only one phosphate and secondary chains of various lengths, numbers, and/or configurations. These structure–function relationships have been exploited to produce analogs that are either LPS antagonists or nontoxic adjuvants.

LPS recognition by CD14–MD-2–TLR4 has received intensive study because it initiates the inflammatory response to so many disease-associated Gram-negative bacteria. Less is known about how animals sense their far more abundant flora of strictly anaerobic Gram-negative bacteria, although doing so may be important for establishing beneficial mutualism between bacteria and host [59,60]. Like the tetraacylated LPS of Porphyromonas gingivalis, the pentaacylated monophosphoryl LPS of Bacteroides fragilis seems to be sensed principally by TLR2 [61–63] and can inhibit recognition of mucosal LPS by TLR4 [64,65].

## Conclusions

An immune system that only recognizes pathogens would leave animals vulnerable to the commensal and colonizing microbes that enter subepithelial tissues at sites of microtrauma throughout life [31]. An innate defense that detects and responds to mucosal commensals as well as pathogens is obviously not impenetrable, however; even commensals may induce damaging responses when host defenses are impaired by trauma, cuts, or tubes that provide conduits across epithelia, immunosuppression, or an inherited immune defect [29,66]. An even greater gap in host defense may be exposed when a Gram-negative pathogen evades TLR4 recognition by producing a nonmucosal lipid A.

If the synthesis proposed here is correct, it would not be surprising to learn that other elements of innate immunity also sense commensal microbes. Animals may also have conserved enzymatic mechanisms for extinguishing microbial signals that are sensed via other receptors [67,68].

## Supporting Information

Table S1 Lipid A Structures of Various Gram-Negative Bacterial LPSs (208 KB DOC)

Table S2 GenBank and Ensemble Accession Numbers Used (25 KB DOC)

### Accession Numbers

The SwissProt (http://www.ebi.ac.uk/swissprot) primary accession numbers discussed in this paper are AOAH (P28039), CD14 (P08571), MD-2 (Q9Y6Y9), and TLR4 (000206).

## Abbreviations

AOAH: acyloxyacyl hydrolase
CAMP: cationic antimicrobial peptide
LPS: lipopolysaccharide
