# Supplementary material for: Shield as Signal: Lipopolysaccharides and the Evolution of Immunity to Gram-Negative Bacteria
Source: PLoS Pathog. 2006 Jun 30;2(6):e67. doi: 10.1371/journal.ppat.0020067 (PMC1483240; doi:10.1371/journal.ppat.0020067)
Supplement: Table S1 — (208 KB DOC) [file ppat.0020067.st001.doc]

**TABLE S1 Munford and Varley**

**Lipid A structures of various Gram-negative bacterial lipopolysaccharides**

Diagram: Structure of *E. coli* lipid A. The backbone positions are indicated.

S = usual site of attachment of polysaccharide chain.

R = secondary acyl chain.

More than one structure is often present in a lipid A preparation. When quantitation of the different structures was reported, the most abundant species is given. The reader is referred to the excellent chapter on lipid A acylation by Zähringer, Linder and Rietschel [1] for additional acylation patterns and references.

| Backbone position | **4’- PO_4_** | | **3’** | **3’R** | **2’** | **2’R** | **3** | **3R** | **2** | **2R** | **1- PO_4_** | **Number of acyl chains** | **REF** |
| --- | --- | --- | --- | --- | --- | --- | --- | --- | --- | --- | --- | --- | --- |
| **Mucosal commensals** | | | | | | | | | | | | | |
| *E. coli* | + | | 14-OH | 14 | 14-OH | 12 | 14-OH | -- | 14-OH | -- | + | 6 | [1] |
| *Klebsiella pneumoniae* | + | | 14-OH | 14 | 14-OH | 14 | 14-OH | -- | 14-OH | -- | + | 6 | [2] |
| *Klebsiella oxytoca* | + | | 14-OH | 14 | 14-OH | 12 | 14-OH | -- | 14-OH | -- | + | 6 | [2] |
| *Serratia marcescens* | + | | 14-OH | 14 | 14-OH | 12 | 14-OH | -- | 14-OH | -- | + | 6 | [3] |
| *Proteus mirabilis* | + | | 14-OH | 14 | 14-OH | 14 | 14-OH | -- | 14-OH | -- or 16 | + | 6 or 7 | [3] |
| *Providencia rettgeri* | + | | 14-OH | 14 | 14-OH | 12 | 14-OH | -- | 14-OH | -- | + | 6 | [3] |
| **Mucosal colonizers, pathogens** | | | | | | | | | | | | | |
| *Aeromonas sp.*  (water habitat) | + | | 14-OH | 12 | 14-OH | 12 | 14-OH | -- | 14:OH | -- | + | 6 | [4] |
| *Plesiomonas shigelloides*  (water habitat) | + | | 12-OH | 12:0 | 14-OH | 14:0 | 12-OH | -- | 14-OH | -- | + | 6 | [1,5] |
| *Moraxella* | + | | 12-OH | -- | 12-OH | 10 | 12-OH | 10 | 12-OH | 12 | + | 7 | [6] |
| *Neisseria (meningitidis, gonorrhoeae)* | + | | 12-OH | -- | 14-OH | 12 | 12-OH | -- | 14-OH | 12 | + | 6 | [7,8] |
| *Haemophilus influenzae, ducreyi* | + | | 14-OH | 14 | 14-OH | 14 | 14-OH | -- | 14-OH | -- | + | 6 | [3] |
| *Bordetella bronchiseptica* | + | | 14-OH | 16 | 14-OH | 14 | 12-OH | -- | 14-OH | -- | + | 6  (bvg+) | [9,10] |
| *Campylobacter*  *jejuni* | + | | 14-OH | 16 | 14-OH | 16 | 14-OH | -- | 14-OH | -- | + | 6 | [11] |
| *Shigella flexneri* | + | | 14-OH | 14 | 14-OH | 12 | 14-OH | -- | 14-OH | -- | + | 6 | [12] |
| *Shigella sonnei* | + | | 14-OH | -- | 14-OH | 14 | 14-OH | -- | 14-OH | 12 | + | 6 | [13] |
| *Salmonella typhimurium* | + | | 14-OH | 14 | 14-OH | 12 | 14-OH | -- | 14-OH | -- or 16 | + | 6 or 7 | [14] |
| *Vibrio cholerae O1* (water habitat) | + | | 12-OH | -- | 14-OH | 14 | 12-OH | -- | 14-OH | 14 | + | 6 | [15] |
| *Vibrio cholerae O139* | + | | 12-OH | 12 | 12-OH | 12 | 12-OH | 16 | 14-OH | 14 | ? | 8 | [15] |
| *Chlamydia* | + | | 14 | -- | 20-OH | 18 | 14 | -- | 20-OH | -- | + | 5 | [16] |
| *Helicobacter pylori* | +/- | | -- | -- | 18-OH | 18 | 16-OH | -- | 18-OH | -- | + | 4 (can have 6) | [17] |
| **Non-mucosal animal habitats** | | | | | | | | | | | | | |
| *Legionella pneumophila* | + | | 14-OH | 27, 28 | n-OH | 16 | 14-OH | -- | n-OH | -- | + | 6  (n = 18, 20, 22) | [18] |
| *Francisella tularensis* | -- | | -- | -- | 18-OH | 16 | 18-OH | -- | 18-OH | -- | + | 4 | [19] |
| *Yersinia pestis*  *(21°C)* | + | | 14-OH | 12 | 14-OH | 16:1 | 14-OH | -- | 14-OH | -- | + | 6 | [20] |
| *Yersinia pestis*  *(37°C)* | + | | 14-OH | -- | 14-OH | -- | 14-OH | -- | 14-OH | -- | + | 4 | [20,21] |
| *Coxiella burnetti* | + | | 16 or 15 | -- | 16-OH | -- | 16 | -- | 16-OH | -- | + | 4 | [22] |
| *Leptospira interrogans* | -- | | 12-OH | 12:1 or 14:1 | 16-OH | 12:1 or 14:1 | 12-OH | -- | 16-OH | -- | + | 6 | [23] |
| **Soil, water, plant habitats** | | | | | | | | | | | | | |
| *Burkholderia cepacia* | | + | 14 | -- | 16-OH | 14 | 14-OH | -- | 16-OH | -- | + | 5 | [24] |
| *Burkholderia caryophylli* | | + | 14-OH or -- | -- | 16-OH | 14 | 14-OH | -- | 16-OH | -- | + | 5 | [2] |
| *Chromobacterium violaceum* | | (+) | 10-OH | -- | 12-OH | 12 | 10-OH | -- | 12-OH | 12 | + | 6 | [3] |
| *Enterobacter agglomerans* | | + | 14-OH | 14-OH | 14-OH | 12 | 14-OH | -- | 14-OH | 16 | + | 7 | [25] |
| *Erwinia carotovora* | | + | 14-OH | 12 | 14-OH | 12 | 14-OH | -- | 14-OH | 16 | + | 7 | [26] |
| *Pseudomonas aeruginosa*  (environmental strain) | | + | 10-OH | -- | 12-OH | 12 | -- | -- | 12-OH | 12 | + | 5 | [27] |
| *Pseudomonas reactans* | | + | 10-OH  or -- | -- | 12-OH | 12 or 12-OH | 10-OH | -- | 12-OH | 12 or 12-OH | + | 5 or 6 | [2] |
| *Rhodobacter sphaeroides* | | + | 10-OH | -- | 14-OH | 14:1 | 10-OH | -- | 14 (3  =O) | -- | + | 5 | [28] |
| *Rhizobium etli* | | + | 14-  OH | -- | 14-OH | 28-O-butyrate | -- or 14-OH | -- | 14-OH | -- | + | 4 or 5 | [29] |
| *Sinorhizobium sp.* | | + | 14-OH | -- | 18-OH | 29-OH | -- or 14-OH |  | 18-OH | -- | + | 4 or 5 | [30] |
| *Xanthomonas campestris* | | (+) | 10-OH or 13-OH | 10 or 11 | 10-OH or 13-OH | -- | 10-OH or 12-OH | 10 or 11 | 12-OH | -- | + | 6  (some are methyl-ated) | [31] |

Reference List

1. Zähringer U, Lindner B, Rietschel ET (1999) Chemical Structure of Lipid A: Recent Advances in Structural Analysis of Biologically Active Molecules. In: Brade H, Opal SM, Vogel SN, Morrison DC, editors. Endotoxin in Health and Disease. New York: Marcel Dekker. pp. 93-114.

2. Silipo A, Lanzetta R, Amoresano A, Parrilli M, Molinaro A (2002) Ammonium hydroxide hydrolysis: a valuable support in the MALDI-TOF mass spectrometry analysis of Lipid A fatty acid distribution. J Lipid Res 43: 2188-2195.

3. Takayama K, Qureshi N (1992) Chemical structure of lipid A. In: Morrison DC, Ryan JL, editors. Bacterial Endotoxic Lipopolysaccharides. Boca Raton, FL: CRC Press. pp. 43-66.

4. El Aneed A, Banoub J (2005) Elucidation of the molecular structure of lipid A isolated from both a rough mutant and a wild strain of Aeromonas salmonicida lipopolysaccharides using electrospray ionization quadrupole time-of-flight tandem mass spectrometry. Rapid Commun Mass Spectrom 19: 1683-1695.

5. Lukasiewicz J, Niedziela T, Jachymek W, Kenne L, Lugowski C (2006) Structure of the lipid A-inner core region and biological activity of Plesiomonas shigelloides O54 (strain CNCTC 113/92) lipopolysaccharide. Glycobiology 16: 538-550.

6. Masoud H, Perry MB, Richards JC (1994) Characterization of the lipopolysaccharide of Moraxella catarrhalis. Structural analysis of the lipid A from M. catarrhalis serotype A lipopolysaccharide. Eur J Biochem 220: 209-216.

7. Kulshin VA, Zahringer U, Lindner B, Frasch CE, Tsai CM, Dmitriev BA, Rietschel ET (1992) Structural characterization of the lipid A component of pathogenic Neisseria meningitidis. J Bacteriol 174: 1793-1800.

8. Ellis CD, Lindner B, Anjam Khan CM, Zahringer U, Demarco dH (2001) The Neisseria gonorrhoeae lpxLII gene encodes for a late-functioning lauroyl acyl transferase, and a null mutation within the gene has a significant effect on the induction of acute inflammatory responses. Mol Microbiol 42: 167-181.

9. Preston A, Maxim E, Toland E, Pishko EJ, Harvill ET, Caroff M, Maskell DJ (2003) Bordetella bronchiseptica PagP is a Bvg-regulated lipid A palmitoyl transferase that is required for persistent colonization of the mouse respiratory tract. Mol Microbiol 48: 725-736.

10. Zarrouk H, Karibian D, Bodie S, Perry MB, Richards JC, Caroff M (1997) Structural characterization of the lipids A of three Bordetella bronchiseptica strains: variability of fatty acid substitution. The Journal of Bacteriology 179: 3756-3760.

11. Moran AP (1995) Biological and serological characterization of Campylobacter jejuni lipopolysaccharides with deviating core and lipid A structures. FEMS Immunol Med Microbiol 11: 121-130.

12. D'Hauteville H, Khan S, Maskell DJ, Kussak A, Weintraub A, Mathison J, Ulevitch RJ, Wuscher N, Parsot C, Sansonetti PJ (2002) Two *msbB* genes encoding maximal acylation of lipid A are required for invasive *Shigella flexneri* to mediate inflammatory rupture and destruction of the intestinal epithelium. J Immunol 168: 5240-5251.

13. Bath UR, Kontrohr T, Mayer H (1987) Structure of *Shigella sonnei* lipid A. FEMS Microbiol Lett 40: 189-192.

14. Takayama K, Qureshi N, Mascagni P (1983) Complete structure of lipid A obtained from the lipopolysaccharides of the heptoseless mutant of Salmonella typhimurium. J Biol Chem 258: 12801-12803.

15. Chatterjee SN, Chaudhuri K (2003) Lipopolysaccharides of Vibrio cholerae: I. Physical and chemical characterization. Biochimica et Biophysica Acta (BBA) - Molecular Basis of Disease 1639: 65-79.

16. Rund S, Lindner B, Brade H, Holst O (1999) Structural Analysis of the Lipopolysaccharide from Chlamydia trachomatis Serotype L2. J Biol Chem 274: 16819-16824.

17. Moran AP, Lindner B, Walsh EJ (1997) Structural characterization of the lipid A component of Helicobacter pylori rough- and smooth-form lipopolysaccharides. J Bacteriol 179: 6453-6463.

18. Zahringer U, Knirel YA, Lindner B, Helbig JH, Sonesson A, Marre R, Rietschel ET (1995) The lipopolysaccharide of Legionella pneumophila serogroup 1 (strain Philadelphia 1): chemical structure and biological significance. Prog Clin Biol Res 392: 113-139.

19. Phillips NJ, Schilling B, McLendon MK, Apicella MA, Gibson BW (2004) Novel Modification of Lipid A of Francisella tularensis. Infect Immun 72: 5340-5348.

20. Rebeil R, Ernst RK, Gowen BB, Miller SI, Hinnebusch BJ (2004) Variation in lipid A structure in the pathogenic yersiniae. Mol Microbiol 52: 1363-1373.

21. Kawahara K, Tsukano H, Watanabe H, Lindner B, Matsuura M (2002) Modification of the structure and activity of lipid A in Yersinia pestis lipopolysaccharide by growth temperature. Infect Immun 70: 4092-4098.

22. Toman R, Garidel P, Andra J, Slaba K, Hussein A, Koch M, Brandenburg K (2004) Physicochemical characterization of the endotoxins from Coxiella burnetii strain Priscilla in relation to their bioactivities. BMC Biochemistry 5: 1.

23. Que-Gewirth NLS, Ribeiro AA, Kalb SR, Cotter RJ, Bulach DM, Adler B, Girons IS, Werts C, Raetz CRH (2004) A Methylated Phosphate Group and Four Amide-linked Acyl Chains in Leptospira interrogans Lipid A: THE MEMBRANE ANCHOR OF AN UNUSUAL LIPOPOLYSACCHARIDE THAT ACTIVATES TLR2. J Biol Chem 279: 25420-25429.

24. Silipo A, Molinaro A, Cescutti P, Bedini E, Rizzo R, Parrilli M, Lanzetta R (2005) Complete structural characterization of the lipid A fraction of a clinical strain of B. cepacia genomovar I lipopolysaccharide. Glycobiology 15: 561-570.

25. Wang Y, Cole RB (1996) Acid and base hydrolysis of lipid A from Enterobacter agglomerans as monitored by electrospray ionization mass spectrometry: pertinence to detoxification mechanisms. J Mass Spectrom 31: 138-149.

26. Fukuoka S, Knirel YA, Lindner B, Moll H, Seydel U, Zahringer U (1997) Elucidation of the structure of the core region and the complete structure of the R-type lipopolysaccharide of Erwinia carotovora FERM P-7576. Eur J Biochem 250: 55-62.

27. Ernst RK, Yi EC, Guo L, Lim KB, Burns JL, Hackett M, Miller SI (1999) Specific lipopolysaccharide found in cystic fibrosis airway *Pseudomonas aeruginosa*. Science 286: 1561-1565.

28. Rose JR, Christ WJ, Bristol JR, Kawata T, Rossignol DP (1995) Agonistic and antagonistic activities of bacterially derived *Rhodobacter sphaeroides* lipid A: Comparison with activities of synthetic material of the proposed structure and analogs. Infect Immun 63: 833-839.

29. Vandenplas ML, Carlson RW, Jeyaretnam BS, McNeill B, Barton MH, Norton N, Murray TF, Moore JN (2002) Rhizobium Sin-1 Lipopolysaccharide (LPS) Prevents Enteric LPS-induced Cytokine Production. J Biol Chem 277: 41811-41816.

30. Gudlavalleti SK, Forsberg LS (2003) Structural Characterization of the Lipid A Component of Sinorhizobium sp. NGR234 Rough and Smooth Form Lipopolysaccharide. DEMONSTRATION THAT THE DISTAL AMIDE-LINKED ACYLOXYACYL RESIDUE CONTAINING THE LONG CHAIN FATTY ACID IS CONSERVED IN RHIZOBIUM AND SINORHIZOBIUM SP. J Biol Chem 278: 3957-3968.

31. Silipo A, Molinaro A, Sturiale L, Dow JM, Erbs G, Lanzetta R, Newman MA, Parrilli M (2005) The Elicitation of Plant Innate Immunity by Lipooligosaccharide of Xanthomonas campestris. J Biol Chem 280: 33660-33668.
