# Supplementary material for: Shield as Signal: Lipopolysaccharides and the Evolution of Immunity to Gram-Negative Bacteria
Source: PLoS Pathog. 2006 Jun 30;2(6):e67. doi: 10.1371/journal.ppat.0020067 (PMC1483240; doi:10.1371/journal.ppat.0020067)
Supplement: Table S2 — (25 KB DOC) [file ppat.0020067.st002.doc]

**Munford and Varley, Figure 2 (ON-LINE SUPPLEMENT)**

GenBank and Ensemble accession numbers used:

ENSCAFP00000004833, XP_539519 (*Canis familiaris*),

PB_163407; PB_160607; CN_161691; CN_163078 (*Sus scrofa*)

ENSBTAG00000018119 (*Bos taurus*)

NM_001637 (*Homo sapiens*)

ENSPTRP00000032605, ENSPTRP00000032607, ENSPTRP00000032606, ENSPTRP00000032608 (*Pan troglodytes*)

ENSMMUP00000029318 (*Macaca mulatta*)

AF018173 (*Oryctolagus cuniculus*)

ENSRNOP00000038138, [XP_574041](http://www.ncbi.nlm.nih.gov/entrez/viewer.fcgi?db=protein&val=62663625) (*Rattus norvegicus*)

NM_012054 (*Mus musculus*)

ENSGALG00000012097, XP_418835 (*Gallus gallus*)

XM_791721 (*Strongylocentrotus purpuratus*)

ENSXETG00000000347 (*Xenopus tropicalis*)

XM-639876 (*Dictyostelium discoideum*)

[XM_817632](http://www.ncbi.nlm.nih.gov/entrez/viewer.fcgi?db=protein&val=15140404) (*Trypanosoma brucei brucei*)
